# Several sharp inequalities about the first Seiffert mean

**DOI:** 10.1186/s13660-018-1763-2

**Published:** 2018-07-16

**Authors:** Boyong Long, Ling Xu, Qihan Wang

**Affiliations:** School of Mathematical Sciences, Anhui University, Hefei, China

**Keywords:** Neuman–Sándor mean, Seiffert mean, Logarithmic mean

## Abstract

In this paper, we deal with the problem of finding the best possible bounds for the first Seiffert mean in terms of the geometric combination of logarithmic and the Neuman–Sándor means, and in terms of the geometric combination of logarithmic and the second Seiffert means.

## Introduction

A mean is a function $f: \mathbb{R}_{+}^{2}\rightarrow \mathbb{R}_{+}$ which satisfies

1.1 $$ \min (a,b)\leq f(a,b)\leq \max (a,b),\quad \forall a, b>0. $$

Each mean is reflexive, namely

1.2 $$ f(a,a)=a,\quad\forall a>0. $$

That is also used as the definition of $f(a,a)$.

A mean is symmetric if

1.3 $$ f(a,b)=f(b,a),\quad\forall a, b>0; $$

it is homogeneous (of degree 1) if

1.4 $$ f(ta,tb)=tf(a,b),\quad\forall a, b, t>0. $$

We shall refer here to some symmetric and homogeneous means as follows.

For $a,b > 0$ with $a \ne b$, the Neuman–Sándor mean $M(a,b)$ [16], the first Seiffert mean $P(a,b)$ [18], the second Seiffert mean $T(a,b)$ [19] and the logarithmic mean $L(a,b)$ are defined by

1.5 $$\begin{aligned}& M ( {a,b} ) = \frac{{a - b}}{{2\sinh ^{-1} ( {\frac{{a - b}}{{a + b}}} ) }}, \end{aligned}$$

1.6 $$\begin{aligned}& P ( {a,b} ) = \frac{{a - b}}{2\arcsin ( {\frac{{a - b}}{{a + b}}} ) }, \end{aligned}$$

1.7 $$\begin{aligned}& T(a,b) = \frac{{a - b}}{{2\arctan ( {\frac{{a - b}}{{a + b}}} ) }} , \end{aligned}$$

and

1.8 $$ L(a,b) = \frac{{a - b}}{{\log a - \log b}}, $$

respectively.

Let $M_{p}(a,b) = {(({a^{p}} + {b^{p}})/2)^{1/p}}$ ($p\neq 0$) stand for the *p*th power means. The mean $M_{1}=A$ is the arithmetic mean, and the mean $M_{2}=Q$ is the root-square mean. The geometric mean is given by $G(a,b) = \sqrt{ab}$, but verifying also the property $\lim_{p\rightarrow 0}M_{p}(a,b)=M_{0}(a,b)=G(a,b)$.

As Carlson remarked in [2], the logarithmic mean can be rewritten as

1.9 $$ L(a,b) = \frac{{a - b}}{{2\tanh ^{-1} ( {\frac{{a - b}}{{a + b}}} ) }}, $$

thus the means *M*, *P*, *T* and *L* are very similar. In [16] it is also proven that these means can be defined using the non-symmetric Schwab–Borchardt mean $\mathcal{SB}$ given by

1.10 $$ \mathcal{SB}(a,b)= \textstyle\begin{cases} \frac{\sqrt{b^{2}-a^{2}}}{\cos ^{-1}(a/b)} &\mbox{if } a< b, \\ \frac{\sqrt{a^{2}-b^{2}}}{\cosh ^{-1}(a/b)}&\mbox{if } a>b; \end{cases} $$

see [1]. It has been established in [16] that

1.11 $$ L=\mathcal{SB}(A,G), \qquad P=\mathcal{SB}(G,A),\qquad T=\mathcal{SB}(A,Q), \qquad M=\mathcal{SB}(Q,A). $$

For two means $\mathcal{M}$ and $\mathcal{N}$ we write $\mathcal{M < N}$ if $\mathcal{M}(a,b) < \mathcal{N}(a, b)$ for $\forall a, b>0$, $a\neq b$. It is well known that the inequalities

1.12 $$ G< L< P< A< M< T< Q. $$

Recently, the inequalities for means have been the subject of intensive research. Many remarkable inequalities can be found in the literature [5, 7, 9, 14, 15, 17, 20].

In [6], Costin and Toader presented

1.13 $$ {M_{\log 2/(\log \pi - \log 2)}}(a,b) < T(a,b) < {M_{5/3}}(a,b) $$

holding for all $a,b > 0$ with $a \ne b$.

The following sharp power mean bounds for the first Seiffert mean $P(a,b)$ are given by Jagers in [13]:

1.14 $$ {M_{1/2}}(a,b) < P(a,b) < {M_{2/3}}(a,b) $$

for all $a,b > 0$ with $a \ne b$. Hästö [11] improved the results of [13] and the sharp result was found that

1.15 $$ P(a,b)>{M_{\log 2/\log \pi }}(a,b). $$

In [8, 12], the authors proved that the double inequalities

1.16 $$\begin{aligned}& {\alpha _{1}}P(a,b) + (1 - {\alpha _{1}})T(a,b) < M(a,b) < {\beta _{1}}P(a,b) + (1 - {\beta _{1}})T(a,b), \end{aligned}$$

1.17 $$\begin{aligned}& {P^{{\alpha _{2}}}}(a,b){T^{1 - {\alpha _{2}}}}(a,b) < M(a,b) < {P^{{\beta _{2}}}}(a,b){T^{1 - {\beta _{2}}}}(a,b), \end{aligned}$$

hold for all $a,b > 0$ with $a \ne b$ if and only if ${\alpha _{1}}\geq 1/3,{\beta _{1}}\leq (1/2)\pi (4/\pi -1/\sinh ^{-1}(1))$, ${\alpha _{2}}\geq 1/3$, ${\beta _{2}}\leq {\log (4\log (1+\sqrt{2})/\pi )}/\log 2$.

In [3, 4, 10], the authors proved that the double inequalities

1.18 $$\begin{aligned}& {\alpha _{3}}Q(a,b) + (1 - {\alpha _{3}})A(a,b) < T(a,b) < {\beta _{3}}Q(a,b) + (1 - {\beta _{3}})A(a,b), \end{aligned}$$

1.19 $$\begin{aligned}& {Q^{{\alpha _{4}}}}(a,b){A^{1 - {\alpha _{4}}}}(a,b) < T(a,b) < {Q^{{\beta _{4}}}}(a,b){A^{1 - {\beta _{4}}}}(a,b), \end{aligned}$$

1.20 $$\begin{aligned}& {P^{{\alpha _{5}}}}(a,b){Q^{1 - {\alpha _{5}}}}(a,b) < M(a,b) < {P^{{\beta _{5}}}}(a,b){Q^{1 - {\beta _{5}}}}(a,b), \end{aligned}$$

1.21 $$\begin{aligned}& {\alpha _{6}}P(a,b) + (1 - {\alpha _{6}})Q(a,b) < M(a,b) < {\beta _{6}}P(a,b) + (1 - {\beta _{6}})Q(a,b), \end{aligned}$$

hold for all $a,b > 0$ with $a \ne b$ if and only if ${\alpha _{3}} \le (4 - \pi )/[(\sqrt{2} - 1)\pi ]$, ${\beta _{3}} \ge 2/3$, ${\alpha _{4}} \le 2/3$, ${\beta _{4}} \ge 4 - 2\log \pi /\log 2$, ${\alpha _{5}} \le 1/2$, ${\beta _{5}} \le [2\log (\log (1 + \sqrt{2} ) + \log 2)]/(2\log \pi - \log 2)$ and ${\alpha _{6}} \ge 1/2$, ${\beta _{6}} \le [\pi (\sqrt{2} \log (1 + \sqrt{2} ) - 1)]/[(\sqrt{2} \pi - 2)\log (1 + \sqrt{2} )]$.

The main purpose of this paper is to find the least values *α* and *β* such that the inequalities

1.22 $$ P(a,b) > {L^{\alpha }}(a,b){T^{1 - \alpha }}(a,b) $$

and

1.23 $$ P(a,b) > {L^{\beta }}(a,b){M^{1 - \beta }}(a,b) $$

hold for all $a,b > 0$ with $a \ne b$. Moreover, we find that both upper bounds for $P(a,b)$ are trivial cases. That is to say, the inequalities $P(a,b) < {L^{{\lambda _{1}}}}(a,b){T^{1 - {\lambda _{1}}}}(a,b)$ and $P(a,b) < {L^{{\lambda _{2}}}}(a,b){M^{1 - {\lambda _{2}}}}(a,b)$ hold for all $a,b > 0$ with $a \ne b$ if and only if ${\lambda _{1}}=0$ and ${\lambda _{2}}=0$, which we will address at the end of this paper.

## Lemmas

To establish our main results, we need several lemmas, which we present in this section.

For $x \in (0,1)$, the following power series expansions of the functions $\sin ^{ - 1}(x)$, $\sinh ^{ - 1}(x)$, $\tan ^{-1}(x)$ and ${\tanh ^{ - 1}}(x)$ are presented:

2.1 $$\begin{aligned}& \sin ^{ - 1}(x) = x + \frac{{{x^{3}}}}{6} + \frac{{3{x^{5}}}}{{40}} + \cdots = \sum_{n = 0}^{\infty }{\frac{{(2n)!}}{{(2n + 1){2^{2n}}{{(n!)}^{2}}}}} {x^{2n + 1}}, \end{aligned}$$

2.2 $$\begin{aligned}& \sinh ^{ - 1}(x) = x - \frac{{{x^{3}}}}{6} + \frac{{3{x^{5}}}}{{40}} - \cdots = \sum _{n = 0}^{\infty }{\frac{{{{( - 1)}^{n}}(2n)!}}{{(2n + 1){2^{2n}}{{(n!)}^{2}}}}} {x^{2n + 1}}, \end{aligned}$$

2.3 $$\begin{aligned}& \tan ^{-1}(x) = x - \frac{{{x^{3}}}}{3} + \frac{{{x^{5}}}}{5} - \cdots = \sum _{n = 0}^{\infty }{\frac{{{{( - 1)}^{n}}}}{{2n + 1}}} {x^{2n + 1}}, \end{aligned}$$

2.4 $$\begin{aligned}& {\tanh ^{ - 1}}(x) = x + \frac{{{x^{3}}}}{3} + \frac{{{x^{5}}}}{5} + \cdots = \sum _{n = 0}^{\infty }{\frac{1}{{2n + 1}}} {x^{2n + 1}}. \end{aligned}$$

### Lemma 2.1

*Let* ${f_{1}}(x) = 4(1 + {x^{2}})\sqrt{1 - {x^{2}}}{\tanh ^{ - 1}}(x) \tan ^{-1}(x)$. *Then*

2.5 $$ {f_{1}}(x) < 4{x^{2}} + 2{x^{4}} - \frac{5}{{12}}{x^{6}} $$

*for* $x \in (0,1)$.

### Proof

Let

2.6 $$\begin{aligned}& g(x) = {\tanh ^{ - 1}}(x)\sqrt{1 - {x^{2}}} - \biggl(x - \frac{1}{6}{x^{3}}\biggr), \end{aligned}$$

then

2.7 $$ g'(x) = \frac{1}{{\sqrt{1 - {x^{2}}} }}h(x), $$

where $h(x)=1 - x{\tanh ^{ - 1}}(x) - \sqrt{1 - {x^{2}}} (1 - \frac{1}{2}{x^{2}})$. Noting that, for any $x\in (0,1)$,

2.8 $$\begin{aligned}& \sqrt{1 - {x^{2}}} > 1 - \frac{1}{2}{x^{2}} - \frac{1}{2}{x^{4}}, \end{aligned}$$

2.9 $$\begin{aligned}& {\tanh ^{ - 1}}(x) > x + \frac{1}{3}{x^{3}}, \end{aligned}$$

we can get

2.10 $$\begin{aligned} h(x)&< 1 - x\biggl(x + \frac{1}{3}{x^{3}} \biggr) - \biggl(1 - \frac{1}{2}{x^{2}}\biggr) \biggl(1 - \frac{1}{2}{x^{2}} - \frac{1}{2}{x^{4}}\biggr) \\ &= - \frac{1}{{12}}{x^{4}} - \frac{1}{4}{x^{6}} < 0. \end{aligned}$$

It follows from (2.7) and (2.10) that

2.11 $$ g'(x)< 0 $$

for $x\in (0,1)$. Considering $g(0) = 0$, then we have $g(x)<0$ for $x\in (0,1)$. That is,

2.12 $$ {\tanh ^{ - 1}}(x)\sqrt{1 - {x^{2}}} < x -\frac{1}{6}{x^{3}} $$

for $x\in (0,1)$.

Considering (2.3), we have

2.13 $$\begin{aligned} \tan ^{-1}(x) \bigl(1+x^{2}\bigr)&=x+\sum _{n= 1}^{\infty }{(-1)^{n+1} \frac{2}{(2n-1)(2n+1)}{x^{2n + 1}}} \\ &=x+\frac{2}{3}{x^{3}}-\frac{2}{15}{x^{5}}+ \frac{2}{35}{x^{7}}-\cdots \\ &< x+\frac{2}{3}{x^{3}} \end{aligned}$$

for $x\in (0,1)$.

Therefore, it follows from (2.12) and (2.13) that

2.14 $$\begin{aligned} f_{1}(x)&< 4\biggl(x-\frac{1}{6}{x^{3}} \biggr) \biggl(x+\frac{2}{3}{x^{3}}\biggr) \\ &=4{x^{2}}+2{x^{4}}- \frac{4}{9}{x^{6}} \\ &< 4{x^{2}}+2{x^{4}}-\frac{5}{{12}}{x^{6}} \end{aligned}$$

for $x \in (0,1)$. □

### Lemma 2.2

*Let* ${f_{2}}(x)=3\tan ^{-1}(x)\sin ^{-1}(x)(1+{x^{2}})$, ${f_{3}}(x)=\tanh ^{-1}(x)\sin ^{-1}(x)(1-{x^{2}})$. *Then*

2.15 $$\begin{aligned}& {f_{2}}(x) > 3{x^{2}} + \frac{5}{2}{x^{4}}+ \frac{1}{12}{x^{6}}, \end{aligned}$$

2.16 $$\begin{aligned}& {f_{3}}(x) > {x^{2}} - \frac{1}{2}{x^{4}}- \frac{1}{2}{x^{6}}, \end{aligned}$$

*for* $x \in (0,1)$.

### Proof

Noticing that

2.17 $$\begin{aligned} \sin ^{-1}(x) \bigl(1 + {x^{2}}\bigr) &> \biggl(x + \frac{1}{6}{x^{3}} + \frac{3}{40}{x^{5}} \biggr) \bigl(1 + {x^{2}}\bigr) \\ &= x + \frac{7}{6}{x^{3}} + \frac{29}{120}{x^{5}}+ \frac{3}{40}{x^{7}}, \end{aligned}$$

we obtain

2.18 $$\begin{aligned} {f_{2}}(x) &= 3\tan ^{-1}(x)\sin ^{ - 1}(x) \bigl(1 + {x^{2}}\bigr) \\ &> 3\tan ^{ - 1}(x) \biggl(x + \frac{7}{6}{x^{3}} + \frac{29}{120}{x^{5}} + \frac{3}{40}{x^{7}}\biggr) \\ &= 3\biggl({x^{2}} + \frac{5}{6}{x^{4}} + \frac{19}{360}{x^{6}} + \frac{107}{1260}{x^{8}} \cdots \biggr) \\ &> 3\biggl({x^{2}} + \frac{5}{6}{x^{4}} + \frac{1}{36}{x^{6}}\biggr)= 3{x^{2}} + \frac{5}{2}{x^{4}} + \frac{1}{12}{x^{6}} \end{aligned}$$

for $x \in (0,1)$. Therefore, (2.15) holds.

By (2.1) and (2.4), we have

2.19 $$\begin{aligned} &f_{3}(x) - \biggl(x^{2} - \frac{1}{2}x^{4} - \frac{1}{2}x^{6}\biggr) \\ &\quad > \biggl(x + \frac{1}{3}x^{3}\biggr) \biggl(x + \frac{1}{6}x^{3}\biggr) \bigl(1 - x^{2}\bigr) - \biggl(x^{2} - \frac{1}{2}x^{4} - \frac{1}{2}x^{6}\biggr) \\ &\quad = \frac{1}{{18}}\bigl(x^{6} -x^{8}\bigr) > 0 \end{aligned}$$

for $x \in (0,1)$. Therefore, (2.16) holds. □

### Lemma 2.3

*If* $x \in (0,1)$, *then one has*

2.20 $$\begin{aligned}& {\tanh ^{ - 1}}(x)\sqrt{1 - {x^{4}}} < x + \frac{1}{3}{x^{3}} - \frac{3}{{10}}{x^{5}}, \end{aligned}$$

2.21 $$\begin{aligned}& \sinh ^{ - 1}(x)\sqrt{1 + {x^{2}}} > x + \frac{1}{3}{x^{3}} - \frac{2}{{15}}{x^{5}}. \end{aligned}$$

### Proof

Let

2.22 $$ {g_{1}}(x) = {\tanh ^{ - 1}}(x)\sqrt{1 - {x^{4}}} - \biggl(x + \frac{1}{3}{x^{3}} - \frac{3}{{10}}{x^{5}} \biggr). $$

It follows that

2.23 $$ {g'_{1}}(x) = \frac{{1 + {x^{2}} - 2{x^{3}}\tanh^{ - 1}(x) - (1 + {x^{2}} - \frac{3}{2}{x^{4}})\sqrt{1 - {x^{4}}} }}{{\sqrt{1 - {x^{4}}} }}. $$

Notice that $\sqrt{1 - x} > 1 - \frac{1}{2}x - \frac{1}{2}{x^{2}}$ for $x \in (0,1)$, therefore $\sqrt{1 - {x^{4}}} > 1 - \frac{1}{2}{x^{4}} - \frac{1}{2}{x^{8}}$ for $x \in (0,1)$. Considering ${\tanh ^{ - 1}}(x) > x + \frac{1}{3}{x^{3}} + \frac{1}{5}{x^{5}}$ for $x \in (0,1)$, we have

2.24 $$\begin{aligned} &1 + {x^{2}} - 2{x^{3}} {\tanh ^{ - 1}}(x) - \biggl(1 + {x^{2}} - \frac{3}{2}{x^{4}} \biggr)\sqrt{1 - {x^{4}}} \\ &\quad < 1 + {x^{2}} - 2{x^{3}}\biggl(x + \frac{1}{3}{x^{3}} + \frac{1}{5}{x^{5}}\biggr) - \biggl(1 + {x^{2}} - \frac{3}{2}{x^{4}}\biggr) \biggl(1 - \frac{1}{2}{x^{4}} - \frac{1}{2}{x^{8}}\biggr) \\ &\quad = - {x^{6}}\biggl(\frac{1}{6} + \frac{{13}}{{20}}{x^{2}} - \frac{1}{2}{x^{4}} + \frac{3}{4}{x^{6}} \biggr) < 0 \end{aligned}$$

for any $x \in (0,1)$.

Equation (2.23) and inequality (2.24) lead to ${g'_{1}}(x) < 0$ for any $x \in (0,1)$. Noting that ${g_{1}}(0) = 0$, thus we have ${g_{1}}(x) < 0$ for any $x \in (0,1)$. Inequality (2.20) is proved.

Let

2.25 $$ {g_{2}}(x) = \sinh ^{ - 1}(x)\sqrt{1 + {x^{2}}} - \biggl(x + \frac{1}{3}{x^{3}} - \frac{2}{{15}}{x^{5}} \biggr). $$

Then one has

2.26 $$ {g'_{2}}(x)=\frac{{(-{x^{2}}+\frac{2}{3}{x^{4}})\sqrt{1+{x^{2}}} + x\sinh^{ - 1}(x)}}{{\sqrt{1 + {x^{2}}} }}. $$

Because $\sqrt{1 + {x^{2}}} < 1 + \frac{1}{2}{x^{2}}$ and $\sinh ^{ - 1}(x) > x - \frac{1}{6}{x^{3}}$ for $x\in (0,1)$, it follows that

2.27 $$\begin{aligned} &\biggl( - {x^{2}} + \frac{2}{3}{x^{4}}\biggr)\sqrt{1 + {x^{2}}} + x\sinh ^{ - 1}(x) \\ &\quad > \biggl( - {x^{2}} + \frac{2}{3}{x^{4}}\biggr) \biggl(1 + \frac{1}{2}{x^{2}}\biggr) + x\biggl(x - \frac{1}{6}{x^{3}}\biggr) \\ &\quad = \frac{1}{3}{x^{6}} > 0 \end{aligned}$$

for any $x \in (0,1)$.

Equation (2.26) and inequality (2.27) lead to ${g'_{2}}(x) > 0$ for any $x \in (0,1)$. Note that $g_{2}(0) = 0$. So ${g_{2}}(x) > 0$ for any $x \in (0,1)$. Inequality (2.21) is established. □

### Lemma 2.4

*Let* ${f_{4}}(x)=3\sinh ^{-1}(x)\tanh ^{-1}(x)\sqrt{1- {x^{4}}}$ *and* ${f_{5}}(x)=2\sin ^{-1}(x)\sinh ^{-1}(x)\sqrt{1+ {x^{2}}}$. *Then*

2.28 $$\begin{aligned}& {f_{4}}(x) < 3{x^{2}} + \frac{1}{2}{x^{4}} - \frac{3}{5}{x^{6}}, \end{aligned}$$

2.29 $$\begin{aligned}& {f_{5}}(x) > 2{x^{2}} + {x^{4}} - \frac{1}{{10}}{x^{6}} \end{aligned}$$

*for any* $x \in (0,1)$.

### Proof

Because

2.30 $$ \sinh ^{ - 1}(x) < x - \frac{1}{6}{x^{3}} + \frac{3}{{40}}{x^{5}}, $$

for $x \in (0,1)$, we can get

2.31 $$ \begin{aligned} {f_{4}}(x) &= 3\sinh ^{ - 1}(x){\tanh ^{ - 1}}(x)\sqrt{1 - {x^{4}}} \\ &< 3\biggl(x - \frac{1}{6}{x^{3}} + \frac{3}{{40}}{x^{5}} \biggr) \biggl(x + \frac{1}{3}{x^{3}} - \frac{3}{{10}}{x^{5}} \biggr) \\ &= {x^{2}}\biggl(3 + \frac{1}{2}{x^{2}} - \frac{{101}}{{120}}{x^{4}} + \frac{9}{{40}}{x^{6}} - \frac{{27}}{{400}}{x^{8}}\biggr) \\ &< {x^{2}}\biggl(3 + \frac{1}{2}{x^{2}} - \frac{{101}}{{120}}{x^{4}} + \frac{9}{{40}}{x^{4}}\biggr) \\ &< {x^{2}}\biggl(3 + \frac{1}{2}{x^{2}} - \frac{3}{5}{x^{4}}\biggr)= 3{x^{2}} + \frac{1}{2}{x^{4}} - \frac{3}{5}{x^{6}} \end{aligned} $$

for any $x \in (0,1)$. This is inequality (2.28).

Observe $\sin ^{ - 1}(x) > x + \frac{1}{6}{x^{3}} + \frac{3}{{40}}{x^{5}}$ for $x \in (0,1)$. It follows that

2.32 $$ \begin{aligned} f_{5}(x) &= 2\sin ^{ - 1}(x)\sinh ^{ - 1}(x)\sqrt{1 + {x^{2}}} \\ &> 2\biggl(x + \frac{1}{6}{x^{3}} + \frac{3}{{40}}{x^{5}} \biggr) \biggl(x + \frac{1}{3}{x^{3}} - \frac{2}{{15}}{x^{5}} \biggr) \\ &= {x^{2}}\biggl(2 + {x^{2}} - \frac{1}{{180}}{x^{4}} + \frac{1}{{180}}{x^{6}} - \frac{1}{{50}}{x^{8}} \biggr) \\ &> {x^{2}}\biggl(2 + {x^{2}} - \frac{1}{{180}}{x^{4}} - \frac{1}{{50}}{x^{8}}\biggr) \\ &> {x^{2}}\biggl(2 + {x^{2}} - \frac{1}{{10}}{x^{4}} \biggr) \end{aligned} $$

for any $x \in (0,1)$. Inequality (2.29) holds. □

## Main results

Note that $L(a,b)$, $P(a,b)$, $T(a,b)$ and $M(a,b)$ are symmetric and homogeneous of degree 1. In this section, without loss of generality, we can assume that $a > b$, then $x: = (a - b)/(a + b) \in (0,1)$. We have the following two theorems.

### Theorem 3.1

*The inequality*

3.1 $$ P(a,b) > {L^{\alpha }}(a,b){T^{1 - \alpha }}(a,b) $$

*holds for all* $a,b > 0$ *with* $a \ne b$ *if and only if* $\alpha \ge \frac{3}{4}$.

### Proof

Noting that

3.2 $$ \frac{P(a,b)}{A(a,b)} = \frac{x}{\sin}^{ - 1}(x),\qquad \frac{T(a,b)}{A(a,b)} = \frac{x}{\tan}^{ - 1}(x),\qquad \frac{L(a,b)}{A(a,b)} = \frac{x}{\tanh}^{ - 1}(x), $$

we have

3.3 $$ \frac{{\log [T(a,b)] - \log [P(a,b)]}}{{\log [T(a,b)] - \log [L(a,b)]}} = \frac{{\log [\sin^{ - 1}(x)] - \log [\tan^{ - 1}(x)]}}{{\log [\tanh^{ - 1}(x)] - \log [\tan^{ - 1}(x)]}}. $$

Direct computations lead to

3.4 $$ \lim_{x \to {0^{+} }} \frac{{\log [\sin^{ - 1}(x)] - \log [\tan^{ - 1}(x)]}}{{\log [\tanh^{ - 1}(x)] - \log [\tan^{ - 1}(x)]}} = \frac{3}{4}. $$

Next, we take the logarithm of (3.1) and consider the difference between the convex combination of $\log L(a,b),\log P(a,b) $ and $\log T(a,b)$ as follows:

3.5 $$\begin{aligned} &\frac{3}{4}\log \bigl[L(a,b)\bigr] + \frac{1}{4}\log \bigl[T(a,b)\bigr] - \log \bigl[P(a,b)\bigr] \\ &\quad = \frac{3}{4}\log \biggl[\frac{{L(a,b)}}{{A(a,b)}}\biggr] + \frac{1}{4} \log \biggl[\frac{{T(a,b)}}{{A(a,b)}}\biggr] - \log \biggl[\frac{{P(a,b)}}{{A(a,b)}}\biggr] \\ & \quad = \frac{3}{4}\log \biggl[\frac{x}{{\tanh^{ - 1}(x)}}\biggr] + \frac{1}{4} \log \biggl[\frac{x}{{\tan^{ - 1}(x)}}\biggr] - \log \biggl[\frac{x}{{\sin^{ - 1}(x)}}\biggr] \\ &\quad = \log \bigl[\sin ^{ - 1}(x)\bigr] - \frac{1}{4}\log \bigl[{ \tan ^{ - 1}}(x)\bigr] - \frac{3}{4}\log \bigl[{\tanh ^{ - 1}}(x)\bigr] \\ &\quad : = {D_{\frac{3}{4}}}(x). \end{aligned}$$

It follows that

3.6 $$\begin{aligned} &{D_{\frac{3}{4}}}\bigl({0^{+} }\bigr) = 0, \end{aligned}$$

3.7 $$\begin{aligned} &{D'_{\frac{3}{4}}}(x) = \frac{{{f_{1}}(x) - {f_{2}}(x) - {f_{3}}(x)}}{{4\sin^{ - 1}(x)\tan^{ - 1}(x)\tanh^{ - 1}(x)(1 - {x^{2}})(1 + {x^{2}})}}, \end{aligned}$$

where ${f_{1}}(x)$, ${f_{2}}(x)$, and ${f_{3}}(x)$ are defined as in Lemmas 2.1 and 2.2, respectively. Thus, from Lemmas 2.1 and 2.2 one deduces that

3.8 $$\begin{aligned} &f_{1}(x) - {f_{2}}(x) - {f_{3}}(x)< 4{x^{2}} + 2{x^{4}} - \frac{5}{{12}}{x^{6}} - \biggl(3{x^{2}} + \frac{5}{2}{x^{4}} + \frac{1}{12}{x^{6}}\biggr) - \biggl({x^{2}} - \frac{1}{2}{x^{4}} - \frac{1}{2}{x^{6}}\biggr) \\ &\quad =0. \end{aligned}$$

Therefore, it follows from (3.6)–(3.8) that

3.9 $$ {D_{\frac{3}{4}}}(x) < 0 $$

for $x \in (0,1)$.

According to (3.5) and (3.9), we conclude that $P(a,b) > {L^{\frac{3}{4}}}(a,b){T^{\frac{1}{4}}}(a,b)$ for all $a,b > 0$ with $a \ne b$. Considering $L(a,b)< P(a,b)< T(a,b)$ holds for all $a,b > 0$ with $a \ne b$, we can see that (3.1) holds for all $a,b > 0$ with $a \ne b$ and $\alpha \ge \frac{3}{4}$.

If $\alpha < \frac{3}{4}$, then Eqs. (3.3) and (3.4) imply that there exists $0 < {\sigma _{1}} < 1$ such that $P(a,b) < {L^{\alpha }}(a,b){T^{1 - \alpha }}(a,b)$ for all *a*, *b* with $(a - b)/(a + b) \in (0,{\sigma _{1}})$. The proof is completed. □

### Theorem 3.2

*The inequality*

3.10 $$ P(a,b) > {L^{\beta }}(a,b){M^{1 - \beta }}(a,b) $$

*holds for all* $a,b > 0$ *with* $a \ne b$ *if and only if* $\beta \ge \frac{2}{3}$.

### Proof

Noting that

3.11 $$\begin{aligned}& \frac{{P(a,b)}}{{A(a,b)}} = \frac{x}{{\sin^{ - 1}(x)}},\qquad \frac{{M(a,b)}}{{A(a,b)}} = \frac{x}{{\sinh^{ - 1}(x)}},\qquad \frac{{L(a,b)}}{{A(a,b)}} = \frac{x}{{\tanh^{ - 1}(x)}}, \end{aligned}$$

we have

3.12 $$ \frac{{\log [M(a,b)] - \log [P(a,b)]}}{{\log [M(a,b)] - \log [L(a,b)]}} = \frac{{\log [\sin^{ - 1}(x)] - \log [\sinh^{ - 1}(x)]}}{{\log [\tanh^{ - 1}(x)] - \log [\sinh^{ - 1}(x)]}}. $$

Direct computations lead to

3.13 $$ \lim_{x \to {0^{+} }} \frac{{\log [\sin^{ - 1}(x)] - \log [\sinh^{ - 1}(x)]}}{{\log [\tanh^{ - 1}(x)] - \log [\sinh^{ - 1}(x)]}} = \frac{2}{3}. $$

Let

3.14 $$\begin{aligned} &\frac{2}{3}\log \bigl[L(a,b)\bigr] + \frac{1}{3} \log \bigl[M(a,b)\bigr] - \log \bigl[P(a,b)\bigr] \\ &\quad = \frac{2}{3}\log \biggl[\frac{{L(a,b)}}{{A(a,b)}}\biggr] + \frac{1}{3} \log \biggl[\frac{{M(a,b)}}{{A(a,b)}}\biggr] - \log \biggl[\frac{{P(a,b)}}{{A(a,b)}}\biggr] \\ &\quad = \frac{2}{3}\log \biggl[\frac{x}{{\tanh^{ - 1}(x)}}\biggr] + \frac{1}{3} \log \biggl[\frac{x}{{\sinh^{ - 1}(x)}}\biggr] - \log \biggl[\frac{x}{{\sin^{ - 1}(x)}}\biggr] \\ &\quad = \log \bigl[\sin ^{ - 1}(x)\bigr] - \frac{1}{3}\log \bigl[{ \sinh ^{ - 1}}(x)\bigr] - \frac{2}{3}\log \bigl[{\tanh ^{ - 1}}(x)\bigr] \\ &\quad : = {D_{\frac{2}{3}}}(x). \end{aligned}$$

It follows that

3.15 $$\begin{aligned}& {D_{\frac{2}{3}}}\bigl({0^{+} }\bigr) = 0, \end{aligned}$$

3.16 $$\begin{aligned}& {D'_{\frac{2}{3}}}(x) = \frac{{{f_{4}}(x) - {f_{5}}(x) - {f_{3}}(x)}}{{3\tanh^{ - 1}(x)\sin^{ - 1}(x)\sinh^{ - 1}(x)\sqrt{1 + {x^{2}}} (1 - {x^{2}})}}, \end{aligned}$$

where ${f_{3}}(x)$, ${f_{4}}(x)$, and ${f_{5}}(x)$ are defined as in Lemmas 2.2 and 2.4, respectively. Thus, from Lemmas 2.2 and 2.4 one deduces that

3.17 $$ {f_{4}}(x) - {f_{5}}(x) - {f_{3}}(x)< 3{x^{2}} + \frac{1}{2}{x^{4}} - \frac{3}{5}{x^{6}} - \biggl(2{x^{2}} + {x^{4}} - \frac{1}{{10}}{x^{6}} \biggr) - \biggl({x^{2}} - \frac{1}{2}{x^{4}} - \frac{1}{2}{x^{6}}\biggr)= 0. $$

Therefore, it follows from (3.15)–(3.17) that

3.18 $$ {D_{\frac{2}{3}}}(x) < 0 $$

for any $x \in (0,1)$.

According to (3.14) and (3.18), we conclude that $P(a,b) > {L^{\frac{2}{3}}}(a,b){M^{\frac{1}{3}}}(a, b)$ for all $a,b > 0$ with $a \ne b$. Considering $L(a,b)< P(a,b)< M(a,b)$ holds for all $a,b > 0$ with $a \ne b$, we can see that (3.10) holds for all $a,b > 0$ with $a \ne b$ and $\beta \ge \frac{2}{3}$.

If $\beta < \frac{2}{3}$, then Eqs. (3.12) and (3.13) imply that there exists $0 < {\sigma _{2}} < 1$ such that $P(a,b) < {L^{\beta }}(a,b){M^{1 - \beta }}(a,b)$ for all *a*, *b*, with $(a - b)/(a + b) \in (0,{\sigma _{2}})$. The proof is completed. □

### Remark

Let us show there is no ${\lambda _{1}},{\lambda _{2}} \in (0,1)$ such that $P(a,b) < {L^{{\lambda _{1}}}}(a,b){T^{1 - {\lambda _{1}}}}(a,b)$ and $P(a,b) < {L^{{\lambda _{2}}}}(a,b){M^{1 - {\lambda _{2}}}}(a,b)$ hold for all $a,b > 0$ with $a \ne b$. Firstly, we assume that ${\lambda _{1}} > 0$, then Eq. (3.3) and $\lim _{x \to {1^{-} }} \frac{{\log [\sin^{ - 1}(x)] - \log [\tan^{ - 1}(x)]}}{{\log [\tanh^{ - 1}(x)] - \log [\tan^{ - 1}(x)]}} = 0$ imply that there exists $0<{\sigma _{3}}<1$ such that $P(a,b) > {L^{{\lambda _{1}}}}(a,b){T^{1 - {\lambda _{1}}}}(a,b)$ for all *a*, *b* with $(a - b)/(a + b) \in (1 - {\sigma _{3}},1)$. This in conjunction with the well-known inequality $P(a,b) < T(a,b)$, which is the case of ${\lambda _{1}} = 0$, indicates that $P(a,b) < {L^{{\lambda _{1}}}}(a,b){T^{1 - {\lambda _{1}}}}(a,b)$ if and only if ${\lambda _{1}} =0$. With the same method, we can obtain $P(a,b) < {L^{{\lambda _{2}}}}(a,b){M^{1 - {\lambda _{2}}}}(a,b)$ if and only if ${\lambda _{2} =0}$.

## Conclusion

In the article, we give the best possible bounds for the first Seiffert mean in terms of the geometric combination of logarithmic and the Neuman–Sándor means, and in terms of the geometric combination of logarithmic and the second Seiffert means.
